# Case Report: A tibial spur causing posterior tibial tendon dysfunction — intraoperative dynamic evidence

**DOI:** 10.3389/fsurg.2026.1887657

**Published:** 2026-08-06

**Authors:** Shixin Liu, Xiaopeng Ma, Liting Wei, Guimiao Li, Hecheng Zhou, Hao Zhang, Dehang Liu, Xiangliang Ge, Zhenhui Sun

**Affiliations:** 1 Department of Foot and Ankle Surgery, The First Hospital of Hebei Medical University, Shijiazhuang, Hebei, China; 2 Department of Anorectal Surgery, Second Hospital of Hebei Medical University, Shijiazhuang, Hebei, China; 3 Clinical College of Hebei Medical University, Shijiazhuang, Hebei, China; 4 Department of Orthopedics, The First Hospital of Hebei Medical University, Shijiazhuang, Hebei, China

**Keywords:** ankle sprain, intraoperative dynamic examination, mechanical impingement, posterior tibial tendon dysfunction, tarsal tunnel, tendon repair, tibial spur

## Abstract

Posterior tibial tendon dysfunction (PTTD) is a progressive and disabling condition that is frequently misdiagnosed, particularly in the context of ankle sprain. Distal tibial spurs have been identified as secondary imaging findings in PTTD, with prior studies reporting a significant statistical association between spur presence and high-grade posterior tibial tendon (PTT) tears. However, whether a tibial spur can directly cause PTT injury through mechanical impingement remains unestablished, with no direct evidence or case reports to date. We report the case of a 33-year-old male who presented with persistent medial ankle pain following recurrent ankle sprains, having undergone a prior lateral ligament repair at an outside institution without symptomatic improvement. Physical examination revealed pronounced tenderness along the PTT sheath with a palpable nodular induration, and the patient was unable to complete the single-limb heel rise test. A diagnostic local anesthetic injection into the PTT sheath resulted in approximately 70%–80% pain relief. Computed tomography identified a posteromedial tibial bony prominence in close anatomical proximity to the PTT. Intraoperative passive plantar flexion and dorsiflexion testing under direct visualization demonstrated repetitive frictional abrasion of the PTT by the tibial spur, providing, to our knowledge, the first reported intraoperative dynamic evidence of mechanical PTT impingement by a tibial spur. A combined surgical procedure comprising spur excision, primary PTT repair, and tarsal tunnel reconstruction was performed. At one-year follow-up, the patient demonstrated substantial clinical improvement, with the Visual Analog Scale pain score decreasing from 7 to 1, the American Orthopaedic Foot and Ankle Society ankle-hindfoot score improving from 32 to 80, and successful completion of the single-limb heel rise test with restoration of hindfoot inversion. This case highlights a rare but clinically significant cause of PTT injury and suggests that bony impingement by a tibial spur should be considered in the differential diagnosis of persistent medial ankle pain following ankle sprain. Computed tomography is recommended as a complementary imaging modality to magnetic resonance imaging when bony impingement is suspected.

## 1 Introduction

Posterior tibial tendon dysfunction (PTTD) is one of the most common causes of progressive collapsing foot deformity (PCFD), with a prevalence of at least 10% in adults over 65 years of age (1, 2). As a progressive and disabling condition, PTTD can lead to functional limitations, chronic pain, and muscle weakness, with advanced cases potentially complicated by arthritis and other severe sequelae. Early recognition and intervention are critical to preventing disability — conservative treatment is effective in the earliest stages, whereas advanced disease may require surgical reconstruction or even joint fusion (3). Notably, PTTD often presents insidiously in its early stages, particularly when concurrent with ankle sprain, making misdiagnosis and delayed diagnosis common, and the true prevalence likely substantially higher than reported (4, 5).

The modern understanding of PTTD was established by Johnson and Strom in 1989, who proposed a three-stage classification system based on tendon integrity, hindfoot position, and deformity flexibility; Myerson later expanded this to four stages in 1997 by adding a fourth stage characterized by deltoid ligament insufficiency and tibiotalar joint involvement (6, 7). Clinically, PTTD typically presents with medial ankle pain and swelling, progressive collapse of the medial longitudinal arch, hindfoot valgus, forefoot abduction, and a positive “too many toes” sign on posterior observation. Common causes of posterior tibial tendon (PTT) injury include degenerative changes from cumulative microtrauma, as well as acute trauma such as fractures and ankle sprains, which may result in tendon dislocation, partial tear, or complete rupture. Inflammatory conditions such as rheumatoid arthritis, and metabolic disorders including gout and diabetes mellitus, are also recognized etiologies of PTTD (8). Abnormalities of the retromalleolar groove — such as a shallow or irregular configuration — have been identified as secondary MRI findings in PTTD, suggesting that bony structural changes may contribute to PTT pathology (9). Distal tibial spurs represent one such bony abnormality. Studies have reported a statistically significant association between tibial spurs and PTT tears, yet whether a tibial spur can cause PTT injury remains unestablished, with no direct evidence or case reports to date (10).

We report a case of persistent medial ankle pain following recurrent ankle sprains. Computed tomography (CT) identified a posteromedial tibial bony prominence, and intraoperative passive ankle range-of-motion testing demonstrated abrasion of the PTT by the spur. The patient underwent a combined surgical procedure comprising spur excision, PTT repair, and tarsal tunnel reconstruction, with satisfactory outcomes. This report aims to elucidate the potential mechanism by which a posteromedial tibial spur may serve as a contributing factor to PTTD, to alert clinicians to include bony impingement in the differential diagnosis of persistent medial ankle pain following ankle sprain, and to highlight the diagnostic value of CT in identifying such lesions.

## 2 Case presentation

**Figure 1:**
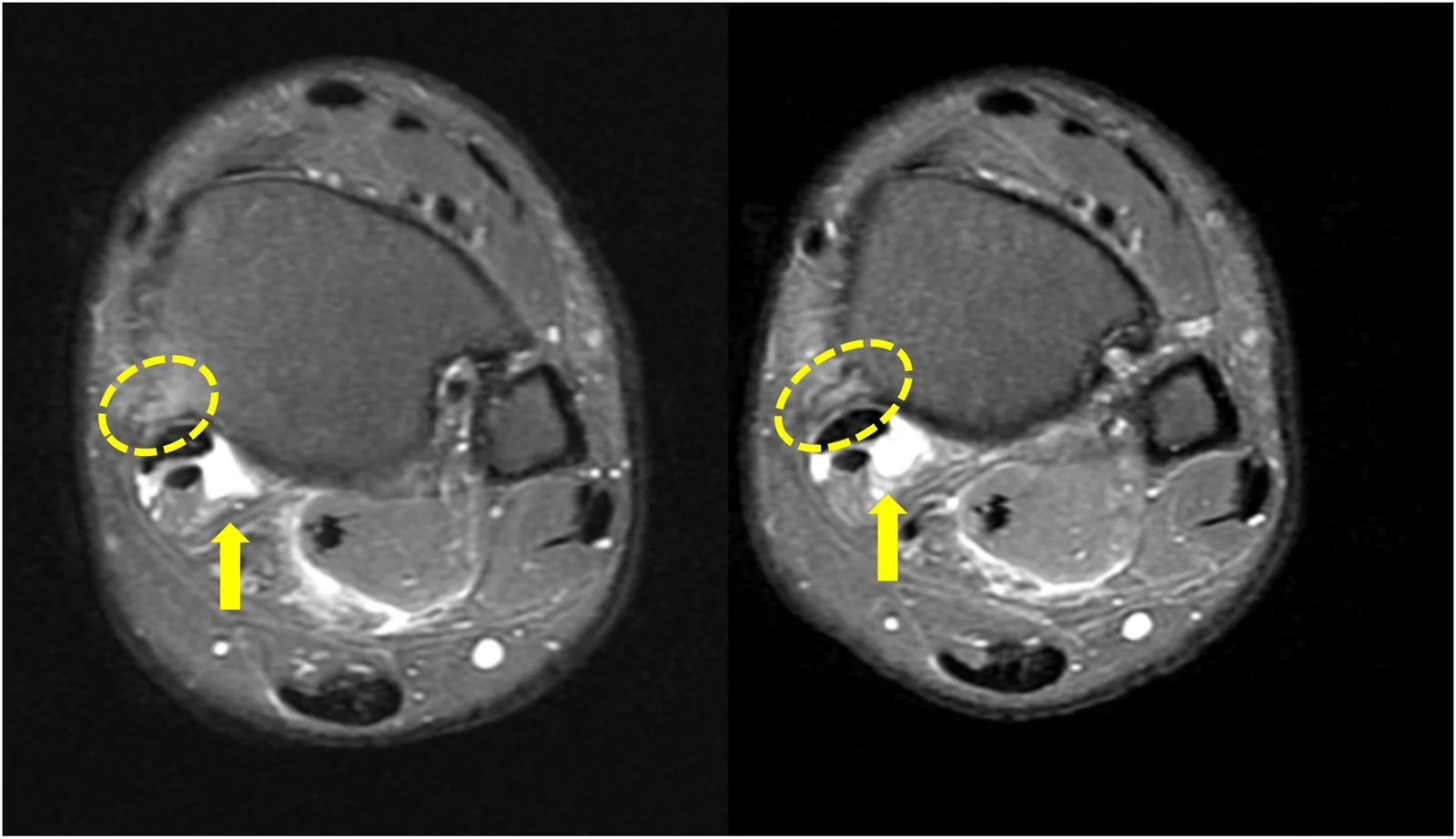
Preoperative MRI of the left ankle obtained approximately five months prior to presentation at our institution. The dashed circle indicates cortical thickening with osteophytic spurring at the posteromedial tibial margin; the yellow arrow identifies peritendinous fluid accumulation.

A 33-year-old male presented with persistent left ankle pain. He had a body mass index (BMI) of 24.5 kg/m², worked as an office clerk involving prolonged sitting, and reported a moderate level of physical activity including regular jogging three times per week. Approximately five months prior to his presentation at our institution, he sustained a mild inversion sprain of the left ankle. Symptoms were minimal and did not interfere with daily activities. MRI obtained at a local hospital at that time demonstrated cortical thickening with osteophytic spurring at the posteromedial tibial margin, along with peritendinous fluid around the PTT sheath. Given the absence of significant symptoms, these findings did not prompt further clinical management (Figure 1).

Approximately three months later, the patient sustained a second, more severe inversion sprain of the left ankle. He developed marked swelling and pain, and was unable to ambulate. He presented to a local hospital and was diagnosed with “left ankle joint injury”, and two weeks later underwent open anterior talofibular ligament imbrication. Following surgery, he remained non-weight-bearing with brace support and crutches. Despite a multimodal analgesic regimen including oral analgesics and physiotherapy, pain persisted without improvement, and he was discharged approximately two weeks postoperatively.

**Figure 2:**
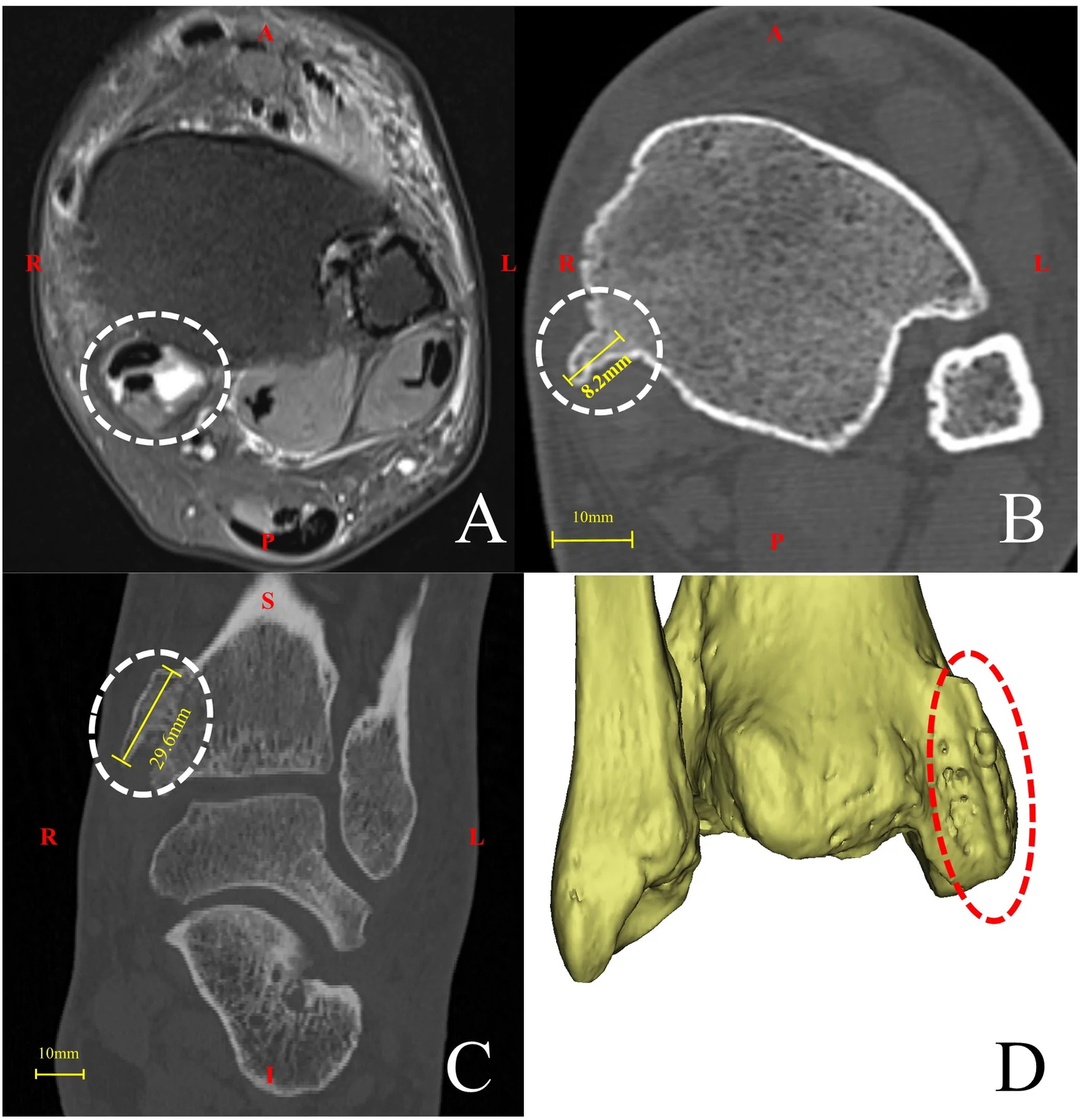
Imaging findings at presentation to our institution. **(A)** MRI of the left ankle demonstrating peritendinous fluid accumulation (dashed circle). **(B)** Axial CT section showing the posteromedial tibial spur (dashed circle). **(C)** Coronal CT section delineating the size and location of the tibial spur (dashed circle). **(D)** Three-dimensional CT reconstruction illustrating the morphology of the tibial spur (dashed circle). (Orientation labels: A, anterior; P, posterior; L, left; R, right; S, superior; I, inferior.).

Approximately three weeks after discharge from the outside institution, the patient presented to our institution with unresolved pain. At presentation, his Visual Analog Scale (VAS) pain score was 7/10 and his American Orthopaedic Foot and Ankle Society (AOFAS) score was 32. Physical examination revealed soft tissue swelling of the left ankle, marked tenderness over the medial malleolus, pronounced tenderness along the PTT sheath with a palpable nodular induration, and no tenderness over the posterior ankle. The patient was unable to complete the single-limb heel rise test, indicating an abnormal result. To objectively localize the primary source of pain, a diagnostic local anesthetic injection was administered into the PTT sheath. Following injection, the VAS score decreased from 7 to 2, representing approximately 70%–80% pain relief. Imaging revealed peritendinous fluid around the PTT on MRI (Figure 2A). CT demonstrated a bony prominence at the posteromedial aspect of the tibia (Figures 2B–D). Based on the integrated clinical and imaging findings, surgical exploration was indicated.

**Figure 3:**
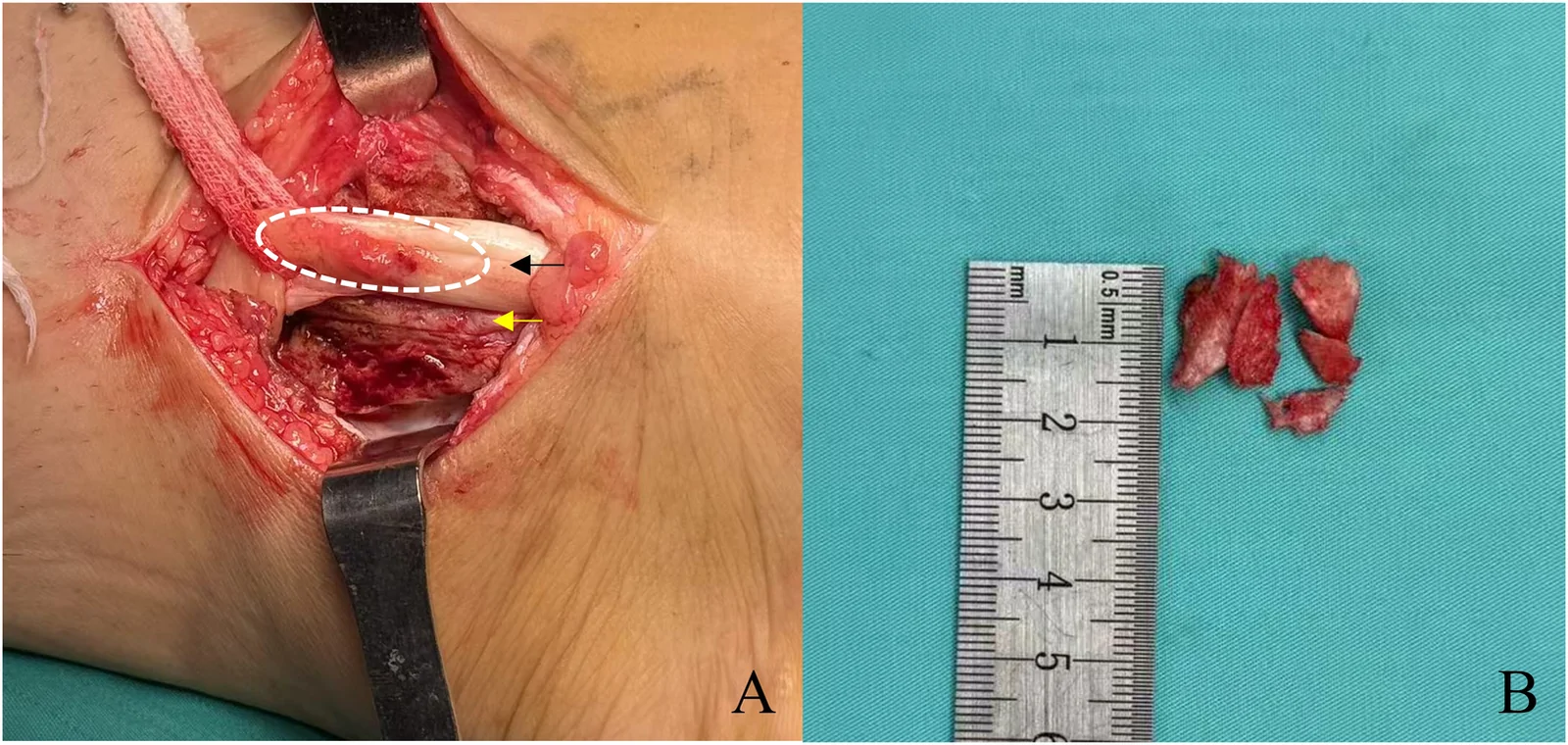
Intraoperative findings. **(A)** Direct visualization of the posterior tibial tendon (black arrow) revealing edema, hyperemia, and surface disruption with degenerative changes (dashed circle) at the site of spur (yellow arrow) contact. **(B)** The excised tibial spur specimen.

The patient was placed in the supine position. A longitudinal incision was made inferior to the medial malleolus, and the tarsal tunnel was exposed through sequential dissection. Blunt dissection of the PTT revealed local edema, hyperemia, and surface disruption upon opening of the tendon sheath (Figure 3A). Passive range-of-motion testing was then performed through plantar flexion and dorsiflexion to simulate the dynamic conditions of gait. Under direct visualization, a bony prominence superior to the tarsal tunnel was observed to produce repetitive frictional contact between the tendon and the spur during ankle motion (Supplementary Video 1). Following confirmation of the pathological mechanism, the PTT tear was repaired with silk suture reinforcement and the tendon was reduced into the tarsal tunnel. The spur was then completely excised using an osteotome, and the bony surface was smoothed with a burr to eliminate the mechanical irritant (Figure 3B). To restore structural integrity of the tarsal tunnel, two suture anchors (Φ3.0 × L11.8 mm; Jiangsu Ihp Medical Co., Ltd., China) were inserted into the medial malleolus, and the anchor sutures were used to reinforce the tarsal tunnel roof in a parallel and cruciate suture configuration, which was tensioned and secured satisfactorily.

**Figure 4:**
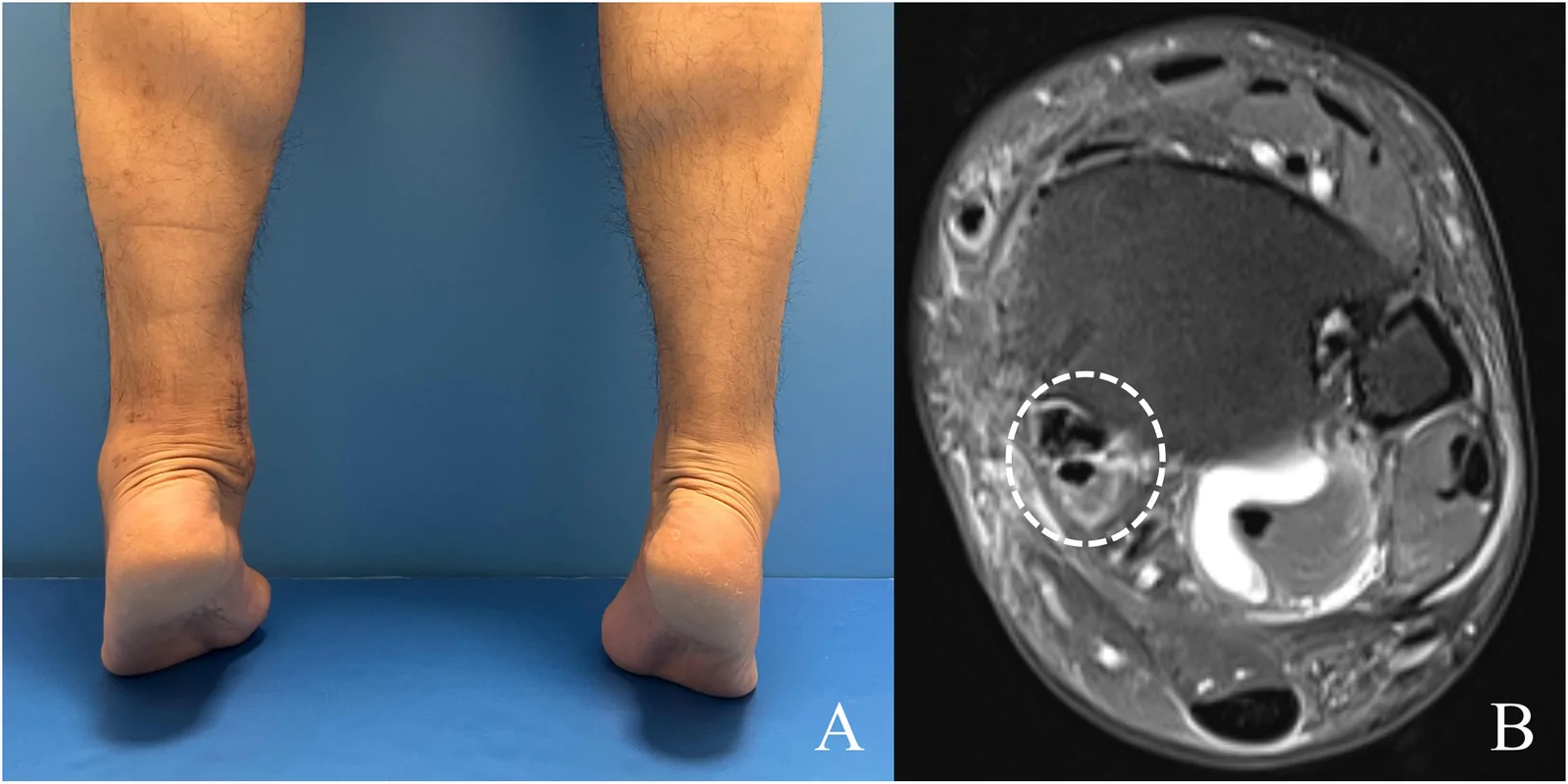
One-year postoperative follow-up. **(A)** Successful completion of the single-limb heel rise test with restoration of hindfoot inversion. **(B)** Follow-up MRI of the ankle showing reduced peritendinous fluid accumulation around the posterior tibial tendon sheath compared to preoperative imaging (dashed circle), consistent with satisfactory surgical outcomes.

Postoperatively, the ankle was immobilized in a short-leg brace for six weeks. Weight-bearing was restricted for the first two weeks, followed by gradual progression to full weight-bearing by six weeks postoperatively. A structured rehabilitation program was initiated at two weeks, comprising range-of-motion exercises, followed by progressive strengthening of the tibialis posterior muscle beginning at six weeks. At discharge, pain was significantly reduced compared to the preoperative level. At one-year follow-up, the patient reported substantial pain relief and had returned to normal ambulation. VAS score was 1/10 and AOFAS ankle-hindfoot score was 80. The single-limb heel rise test was successfully completed with restoration of hindfoot inversion (Figure 4A). Follow-up MRI demonstrated reduction in bone marrow edema of the distal tibia compared to prior imaging (Figure 4B). Although dynamic ultrasound or MRI assessment of tendon integrity was not performed, the reduction in peritendinous fluid signal and the patient's restoration of single-limb heel rise function were considered indicative of satisfactory tendon healing. Both functional scores and imaging findings indicated satisfactory surgical outcomes.

## 3 Discussion

The posterior tibial tendon is the primary stabilizer of the medial longitudinal arch and hindfoot inversion, and its anatomical course confers unique biomechanical vulnerability. The PTT descends from the deep posterior compartment of the leg, curves sharply — approximately 90° — around the posterior aspect of the medial malleolus, and inserts onto the navicular and tarsal bones. This acute angulation subjects the tendon to substantial tensile and frictional forces at the retromalleolar groove (11). Under normal conditions, the retromalleolar groove has a shallow concave configuration with a mean depth of approximately 1.6 mm, providing bony guidance and protection for the PTT (12); the flexor retinaculum, extending from the medial malleolus to the calcaneal, acts in concert with the groove to maintain the tendon in position (13). Any alteration in the bony anatomy will adversely affect the mechanical environment of the PTT. Furthermore, a hypovascular zone exists at the level of the medial malleolus, extending from approximately 2.2 cm proximal to 0.6 cm proximal to the medial malleolus, with a mean length of approximately 1.5 cm. The vascular volume in this region is only approximately 15 μ L/g of tendon tissue, significantly lower than in the proximal and distal portions of the tendon (14). The absence of a mesotenon and the presence of this hypovascular zone render this segment highly susceptible to mechanical stress — repeated friction or impingement at this location readily precipitates inflammation, degeneration, and tearing.

Distal tibial spurs have been recognized as a secondary sign of PTTD. Oddy et al. found that spurs were present in 63.4% of patients with high-grade partial or complete PTT tears, compared with only 3.9% in the non-PTTD control group, a statistically significant association (OR = 4.98, *p* < 0.001) (10). However, this study can only establish a statistical association between spur presence and PTT injury; it cannot determine whether the spur is a secondary consequence of chronic PTT inflammation or an active mechanical impingement factor. These two mechanisms are not mutually exclusive. Biologically, cumulative PTT microtrauma may induce periosteal inflammation at the posteromedial tibia, thereby promoting spur formation (15). From a tribological perspective, the posteromedial tibial spur functions as a rigid, irregular surface that disrupts the normally smooth gliding interface within the tarsal tunnel, producing repetitive mechanical abrasion and contact pressure during ankle motion. Such cyclic mechanical overload disrupts collagen fibril integrity and activates tenocytes to upregulate matrix metalloproteinases — particularly MMP-1 and MMP-3 — accelerating extracellular matrix degradation and impairing intrinsic tendon repair (16–18). This degenerative process is further amplified by the inherent vulnerability of the retromalleolar PTT segment, where vascular volume averages only 15 μ L/g of tendon tissue, severely limiting the local capacity for repair and regeneration (14). In the present case, passive ankle range-of-motion testing under direct visualization demonstrated dynamic frictional abrasion of the PTT by the tibial spur, and symptom resolution following spur excision provided, to our knowledge, the first reported intraoperative evidence supporting the hypothesis of PTT impingement by the tibial spur. Histopathological examination of the peritendinous tissue demonstrated chronic inflammation, consistent with longstanding mechanical irritation at the spur–tendon interface. Pathological analysis of the bony spur was not performed; however, the intraoperative morphology and CT characteristics were consistent with a reactive osteophyte rather than a neoplastic or infectious process.

The mechanical impingement pattern observed in this case shares conceptual similarities with previously reported cases of tendon injury caused by bony structural abnormalities. Gkoudina et al. reported a case of PTT dislocation attributable to retromalleolar groove dysplasia, in which an abnormal bony configuration disrupted the normal mechanical environment of the PTT, resulting in tendon displacement and injury (13). While both cases involve bony anatomical abnormality as the primary pathological driver, the mechanism differs: groove dysplasia results in loss of bony constraint and tendon dislocation, whereas a posteromedial tibial spur introduces an aberrant contact surface that generates frictional abrasion against the tendon. These cases suggest that bony structural abnormalities represent an underrecognized but clinically significant category of tendon injury mechanisms that warrants greater attention in clinical practice.

The contrasting clinical outcomes of the two sprain episodes in this patient — minimal symptoms after the first sprain versus persistent PTT injury after the second sprain — suggest that a spur alone is insufficient to cause injury and may remain asymptomatic. The tarsal tunnel is a confined space with a flexor retinaculum of very limited distensibility. The spur, as a chronic space-occupying lesion, preemptively reduces the available space within the tarsal tunnel. When a more severe ankle sprain triggers acute inflammatory edema, the intra-tunnel pressure exceeds the compensatory threshold, further compressing the interval between the PTT and the spur, and converting dynamic friction into mechanical impingement injury (19).

The two episodes of missed diagnosis prior to the patient's presentation at our institution do not represent isolated oversights, but rather reflect systemic deficiencies in the clinical recognition of PTTD. Epidemiological data indicate that PTTD prevalence is substantially underreported: Kohls-Gatzoulis et al. found a prevalence of 3.3% among women over 40 years of age, with all affected individuals remaining undiagnosed despite typical and persistent symptoms (1). Durrant et al. attributed the high rate of PTTD misdiagnosis to three factors: insufficient specialist referral, inadequate disease awareness among non-specialist clinicians, and the absence of standardized diagnostic criteria (20). These challenges are compounded in the context of ankle sprain, where existing clinical frameworks are primarily oriented toward the evaluation of lateral ligament injury. The Ottawa Ankle Rules, the most widely applied clinical decision tool for acute ankle sprain, are designed exclusively to exclude fractures and have no evaluative capacity for medial soft tissue structures such as the PTT — even a complete PTT tear would not trigger further imaging evaluation if the patient is able to bear weight and has no bony tenderness (21). These systematic blind spots make PTT injury highly susceptible to being overlooked in the setting of ankle sprain. In the present case, both prior clinical encounters were framed around “ankle sprain,” and the medial structures were never systematically assessed. For patients with persistent medial ankle pain following ankle sprain, clinicians should include PTT pathology in the differential diagnosis and perform a systematic examination including palpation of the PTT sheath and single-limb heel rise testing. MRI effectively characterizes the nature and extent of PTT injury, while CT offers superior delineation of bony anatomical abnormalities (22). Clinicians should consider CT when MRI demonstrates PTT signal abnormality without an identifiable intrinsic tendon cause, or when physical examination suggests a space-occupying lesion within the tarsal tunnel. In this case, MRI identified peritendinous fluid but did not delineate the tibial spur with sufficient clarity to characterize its morphology or guide surgical planning; CT provided precise three-dimensional characterization of spur size and location that proved critical for preoperative decision-making.

The surgical strategy in this case comprised spur excision, PTT repair, and tarsal tunnel reconstruction. Literature on the surgical management of tarsal tunnel compression by bony exostoses emphasizes that delayed decompression may result in irreversible functional deficits (23). Regarding PTT repair, intraoperative findings of partial tearing with degeneration were consistent with stage II PTTD. For cases with partial tearing, primary repair of degenerated tissue is a reasonable approach, with good to excellent outcomes when performed in the acute setting (24). Tarsal tunnel reconstruction represented the most innovative component of this surgical strategy. The flexor retinaculum serves not only as the roof of the tarsal tunnel but also as the primary restraint maintaining the PTT within the retromalleolar groove. Anatomical studies have demonstrated that the flexor retinaculum has very limited capacity for spontaneous healing following division, with the gap potentially expanding rather than closing at three months postoperatively — suggesting that simple release without repair risks loss of tendon constraint (25). Following spur excision and PTT repair, suture anchors were inserted into the medial malleolus and the anchor sutures were used to reattach and reinforce the tarsal tunnel roof, simultaneously achieving decompression and structural reconstruction of the tarsal tunnel. This combined approach has no precedent in the literature, and its long-term efficacy warrants evaluation in larger series.

This report is subject to several inherent limitations. As a single case report, the level of evidence is limited; the causal relationship between the tibial spur and PTT injury cannot entirely exclude the contributions of traumatic injury from the ankle sprain or intrinsic tendon degeneration as confounding factors, and prospective studies with larger sample sizes are required for confirmation. The long-term outcomes of the tarsal tunnel reconstruction component also require extended follow-up observation. Additionally, the absence of a preoperative photograph documenting the failed single-limb heel rise test represents a limitation.

Future research directions suggested by this case include: longitudinal investigation of the relationship between retromalleolar groove morphological abnormalities and spur formation; prospective studies designed to clarify the temporal sequence between posteromedial tibial spurs and PTT injury; the development of standardized diagnostic pathways incorporating CT imaging and systematic PTT assessment for patients with persistent medial ankle pain following ankle sprain; and multi-case series and controlled studies to validate the efficacy and safety of the combined surgical approach of spur excision, PTT repair, and tarsal tunnel reconstruction.

## 4 Conclusion

We report a rare case of persistent medial ankle pain following recurrent ankle sprains. CT identified a posteromedial tibial spur, and intraoperative ankle range-of-motion testing provided direct visualization of spur-induced PTT abrasion, representing, to our knowledge, the first reported intraoperative evidence for mechanical PTT impingement by a tibial spur as a pathological mechanism. This case suggests that a posteromedial tibial spur may serve as a rare contributing factor to PTTD. In patients presenting with persistent medial ankle pain following ankle sprain, bony impingement should be included in the differential diagnosis, with systematic physical examination and combined MRI and CT imaging where indicated. A combined surgical approach of spur excision, PTT repair, and tarsal tunnel reconstruction represents an effective strategy for managing this condition, with satisfactory functional and imaging outcomes at one-year follow-up. The precise causal relationship between tibial spurs and PTT injury warrants confirmation through prospective studies with larger patient cohorts.

## Data Availability

The original contributions presented in the study are included in the article/Supplementary Material, further inquiries can be directed to the corresponding author.
